# DNA demethylation by 5-aza-2′-deoxycytidine is imprinted, targeted to euchromatin, and has limited transcriptional consequences

**DOI:** 10.1186/s13072-015-0004-x

**Published:** 2015-03-17

**Authors:** María-Paz Ramos, Neil Ari Wijetunga, Andrew S McLellan, Masako Suzuki, John M Greally

**Affiliations:** Center for Epigenomics and Department of Genetics, Albert Einstein College of Medicine, 1031 Morris Park Avenue, Price 322, Bronx, NY 10461 USA

## Abstract

**Background:**

DNA methylation can be abnormally regulated in human disease and associated with effects on gene transcription that appear to be causally related to pathogenesis. The potential to use pharmacological agents that reverse this dysregulation is therefore an attractive possibility. To test how 5-aza-2′-deoxycytidine (5-aza-CdR) influences the genome therapeutically, we exposed non-malignant cells in culture to the agent and used genome-wide assays to assess the cellular response.

**Results:**

We found that cells allowed to recover from 5-aza-CdR treatment only partially recover DNA methylation levels, retaining an epigenetic ‘imprint’ of drug exposure. We show very limited transcriptional responses to demethylation of not only protein-coding genes but also loci-encoding non-coding RNAs, with a limited proportion of the induced genes acquiring new promoter activation within gene bodies. The data revealed an uncoupling of DNA methylation effects at promoters, with demethylation mostly unaccompanied by transcriptional changes. The limited panel of genes induced by 5-aza-CdR resembles those activated in other human cell types exposed to the drug and represents loci targeted for Polycomb-mediated silencing in stem cells, suggesting a model for the therapeutic effects of the drug.

**Conclusions:**

Our results do not support the hypothesis of DNA methylation having a predominant role to regulate transcriptional noise in the genome and indicate that DNA methylation acts only as part of a larger complex system of transcriptional regulation. The targeting of 5-aza-CdR effects with its clastogenic consequences to euchromatin raises concerns that the use of 5-aza-CdR has innate tumorigenic consequences, requiring its cautious use in diseases involving epigenetic dysregulation.

**Electronic supplementary material:**

The online version of this article (doi:10.1186/s13072-015-0004-x) contains supplementary material, which is available to authorized users.

## Background

With the increasing recognition that disturbances in DNA methylation (5-methylcytosine (5mC)) occur in a variety of human diseases, attention is focusing on how these insights could translate into therapeutic approaches. The field of epigenetic therapeutics has its foundations in cancer biology [1], but the recognition that epigenetic regulatory mechanisms appear to be contributing to diseases other than cancer has prompted discussion of the use of these agents in a broader spectrum of diseases [2]. Targets for epigenetic therapies include DNA methylation and post-translational modifications of histones, including acetylation and methylation, by targeting the enzymes that add these covalent marks. As DNA methylation is currently the best studied of all candidate epigenetic regulators in human diseases, much attention has focused on DNA methyltransferase (DNMT) inhibitors. Several agents have been described to act as DNMT inhibitors: the nucleoside inhibitors 5-azacytidine (5-aza-CR), 5-aza-2′-deoxycytidine (5-aza-CdR), and zebularine; the non-nucleoside inhibitors procaine, epigallocatechin-3-gallate (EGCG), and hydralazine; and the direct DNMT inhibitor RG108 [3,4]. Of these, 5-aza-CdR (decitabine) has been found to be the most effective at demethylating DNA [3] and is approved for the treatment of myelodysplastic syndrome (MDS) in human subjects.

Incorporation of 5-aza-CdR into the genome causes it to be recognized by mammalian DNMT1 which becomes irreversibly bound to the nucleoside, unable to perform its catalytic functions, and leads it to become prematurely degraded, potentially involving ubiquitin-dependent proteasomal degradation [5]. The demethylation of the genome, especially in promoter regions, is a goal of oncological therapy, prompted by observations of the acquisition of DNA methylation at transcription start sites and the associated transcriptional silencing of tumor-suppressor genes [6]. Resistance to 5-aza-CdR has been found to involve differences in rates of incorporation of the nucleoside into DNA [7]. We have previously found that CD34+ hematopoietic stem and progenitor cells (HSPCs) from patients with MDS have distinctive DNA methylation patterns when compared with CD34+ HSPCs from control subjects and that treatment with 5-aza-CR induces loss of DNA methylation at promoters in these cells [8]. In cell models of leukemia, genomic studies have indicated that 5-aza-CR and 5-aza-CdR both induce demethylation of CG dinucleotide-rich CpG islands at promoters, but these promoter changes are not associated with transcriptional effects at those genes [9]. We have also previously observed that long-term hematopoietic stem cells (HSCs, lineage^−^/CD34+/CD38-/CD90+) in MDS have abnormal DNA methylation compared with the same cell type from healthy control subjects and that treatment with 5-aza-CR does not influence the levels of mosaicism for cytogenetic abnormalities in these HSCs, indicating that the therapeutic response is through effects on the functional properties of these neoplastic cells rather than their eradication [10]. A major concern with the use of DNMT inhibitors is their potential to induce genomic rearrangements, traditionally attributed to global demethylation based on cytogenetic observations made in the immunodeficiency, centromeric region instability, facial anomalies (ICF) syndrome [11] but also attributable to the formation of DNMT1 adducts in cells treated by 5-aza-CdR [12].

The genomic response to DNMT inhibitors is one of global demethylation, but there is some heterogeneity of response of loci within the genome. Among the regions undergoing demethylation, some lose while others retain nucleosomal occupancy [13], indicating that transcriptional regulatory processes are not primarily driven by DNA methylation and can be decoupled. With advances in technologies that allow genome-wide studies of DNA methylation, chromatin constituents, and transcription, we now have greater opportunity for more extensive insights into the effects of DNMT inhibitors than prior studies, which tended to focus on gene promoter effects. In particular, we were interested in following up on a prior paradoxical observation that demonstrated DNA methylation to be enriched at DNase hypersensitive, early-replicating euchromatin in non-cancer cell lines [14], suggesting that the effects of a DNA demethylating agent would be more likely to target this gene- and transcription-rich genomic compartment. Another question raised was whether global DNA demethylation is causally associated with the emergence of cryptic promoters. This idea was prompted by a long-established hypothesis that DNA methylation evolved to allow the suppression of transcriptional noise as genome sizes expanded, helping to prevent spurious polymerase-DNA interactions [15]. With these questions in mind, we performed a study to test how 5-aza-CdR influences DNA methylation, RNA polymerase II localization, and gene expression in cultured human embryonic kidney (HEK) 293 T cells. As our previous studies were performed in primary cell types [8,10], our preference was to avoid using the cell lines derived from malignant cells that are typically used in DNMT1 inhibitor studies. However, to allow our studies to be reproduced, we chose to use the commonly used HEK 293 T cell line which is transformed but not derived from cancer cells.

## Results

### Imprinted global demethylation following 5-aza-CdR treatment

**Figure 1:**
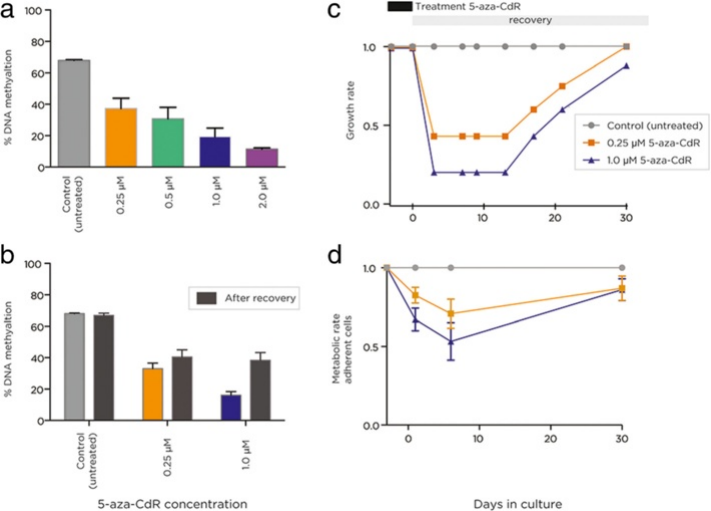
**Dose-dependent and imprinted demethylating effects of 5-aza-CdR.** In panel **(a)**, we show that global DNA methylation measured by LUMA is strongly depleted in a dose-dependent manner following 3 days of 5-aza-CdR exposure. In panel **(b)**, we show that DNA methylation does not return to pre-exposure levels following a month of recovery in tissue culture despite **(c)** a return to pre-treatment cell growth rates (normalized to the untreated cells) and **(d)** a near-complete recovery of cell metabolic rate (using the WST-1 assay). 5-aza-CdR, 5-aza-2′-deoxycytidine.

To test the effect of global demethylation of the genome, we treated the HEK 293 T cell line with a range of 5-aza-CdR concentrations. We chose HEK 293 T cells as a non-cancer cell type that is widely available, allowing replication of our studies and an insight into effects of DNMT1 inhibitors in cells that have not undergone malignant transformation. After 72 h of constant exposure to the drug, we measured global 5mC levels by performing the Luminometric Methylation Assay (LUMA) [16]. The level of 5mC in untreated HEK 293 T cells was approximately 68% but decreased strongly and in a dose-dependent manner following 72 h of treatment (Figure 1a). Focusing in the 0.25 and 1.0 μM 5-aza-CdR-treated cells, conditions that showed striking changes in DNA methylation, we allowed the treated cells to recover and grow in culture for a further 30 days, observing that the growth rates return to pre-treatment levels while cellular metabolic rates recover almost completely during this period (Figure 1c, d). However, further LUMA measurements of total 5mC showed that this return to normal growth was accompanied by only a partial reacquisition of DNA methylation (Figure 1b). This re-setting of the epigenome represents a pharmacologically induced ‘imprinting’ of global DNA methylation levels in these cultured cells.

### DNA demethylation by 5-aza-CdR is predominantly targeted to euchromatin

**Figure 2:**
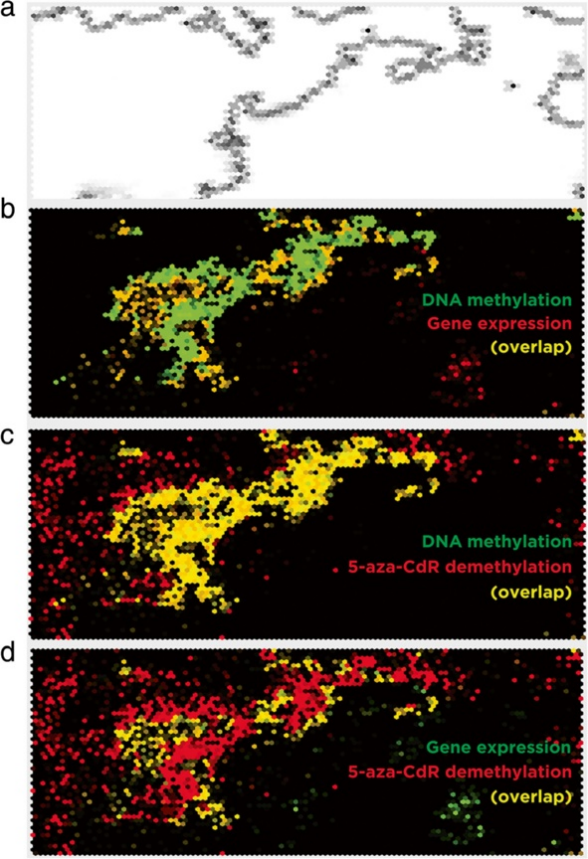
**Demethylation of the genome by 5-aza-CdR predominantly targets euchromatin.** A self-organizing map (SOM) analysis shows clear partitioning of the human genome into distinct compartments, represented by the U matrix of panel **(a)**. This depiction shows increased levels of gray for nodes within the SOM where there is dissimilarity between adjacent nodes in terms of genomic characteristics, illustrating the structure of the partitioning of the information contained within the SOM. In panel **(b)** we see that the left partition includes the co-localization (yellow) of the highest quantiles of DNA methylation (green) and of gene expression (red), loci we previously showed to represent the euchromatic compartment of the genome [14]. When we overlay the loci with the greatest changes in DNA methylation resulting from the 1.0 μM 5-aza-CdR exposure (red), we see enrichment at the euchromatic compartment represented by the loci with the highest DNA methylation levels (panel **(c)**, green) or gene expression levels (panel **(d)**, green). 5-aza-CdR, 5-aza-2′-deoxycytidine.

We then asked whether the 5-aza-CdR treatment affected some genomic contexts to a greater extent than others. We used massively parallel sequencing of ribonucleic acid (RNA-seq) and DNA methylation assay data from untreated control cells to define the euchromatic and heterochromatic compartments of the HEK 293 T genome, using an approach that we have previously published [14]. In that prior study, we showed the most transcriptionally active loci in the genome to be those at which DNA methylation was at its most enriched, regions which were early replicating and relatively DNase hypersensitive, defining them as euchromatic [14]. We reproduced the same analytical approach of our prior study which had used different cell lines (lymphoblastoid cells and fibroblasts) and showed that, using the same self-organizing map (SOM) approach, the genome could be compartmentalized (Figure 2a) and that DNA methylation is enriched at areas of the highest transcriptional activity (Figure 2b). We then tested whether this euchromatic compartment of HEK 293 T cells was related to where we saw the greatest degree of DNA demethylation with 5-aza-CdR treatment, confirming this expectation as shown in Figure 2c, d. As a guide to these regions, we list the Reference Sequence (RefSeq) genes that are in these genomic windows with the highest quantiles of gene expression and of DNA methylation in Additional file 1: Table S1.

### Limited transcriptional effects of DNA demethylation by 5-aza-CdR

**Figure 3:**
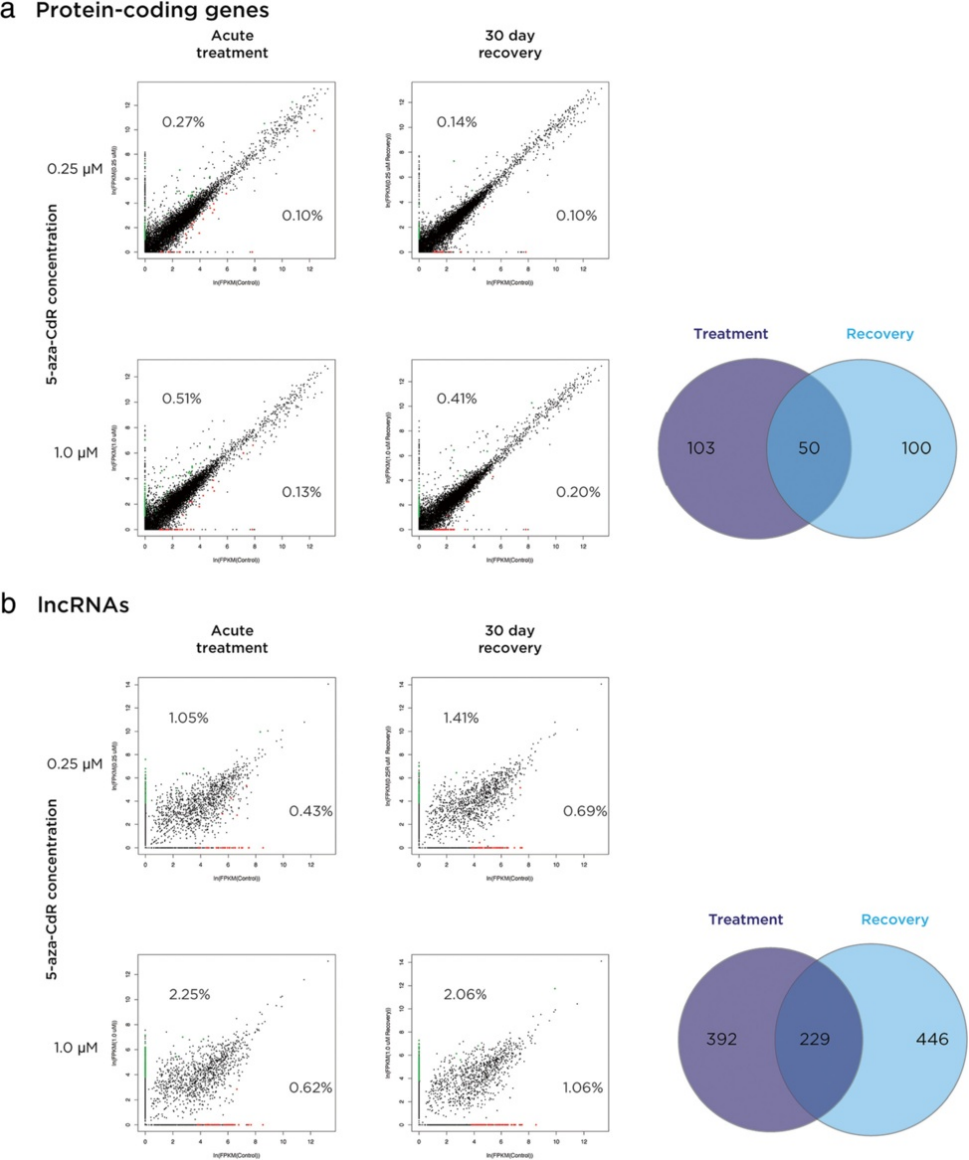
**Limited transcriptional consequences of 5-aza-CdR-mediated genomic demethylation.** We show protein-coding **(a)** and long non-coding RNA **(b)** genes to have very limited proportions of genes that are up-regulated (green) or down-regulated (red) in exposed or recovered cells. The proportion of genes in each situation is indicated. The x axis in each case is control cells not exposed to 5-aza-CdR at that time point. FPKM, fragments per kilobase per million reads; lncRNA, long non-coding RNA; 5-aza-CdR, 5-aza-2′-deoxycytidine.

Having shown demethylation due to 5-aza-CdR exposure to be targeting the most transcriptionally rich compartment of the genome, we wanted to test whether this targeting was reflected by any quantitative or qualitative changes in gene expression. We therefore performed a directional RNA-seq protocol that is not restricted to representing transcripts with a polyA tail (adapted from [17]) and tested the untreated and treated cell samples to test for transcriptional changes using *cufflinks* [18]. As all four conditions (two doses and two time points) were generating highly comparable outcomes of profound demethylation, we used the four conditions as biological replicates, as recommended by the Encyclopedia of DNA Elements (ENCODE) consortium [19]. Expression changes for both protein-coding and long non-coding RNAs were extremely limited in terms of the proportions of genes affected (Figure 3 and Additional file 1: Tables S1 and S2), involving no more than approximately 1% of protein-coding genes and approximately 3% of long non-coding RNAs (lncRNAs). Focusing on the 1.0 μM acute treatment, which had the most substantial effects in terms of the number of genes altered in terms of expression levels, we found that of the 179 up-regulated protein-coding genes, 104 of these were annotated to have multiple RefSeq isoforms, as had 37 of the 72 down-regulated genes, leaving open the possibility that promoter and/or splicing differences were associated with the altered expression of these subgroups of genes. Of the genes induced to change expression with acute 1.0 μM 5-aza-CdR treatment, 19.76% (50 genes) of the differentially expressed protein-coding genes and 17.67% (229 transcripts) of the differentially expressed lncRNAs remain altered in transcription following 30 days of recovery (Figure 3). The genes changing expression are listed in Additional file 1: Tables S1 and S2.

**Figure 4:**
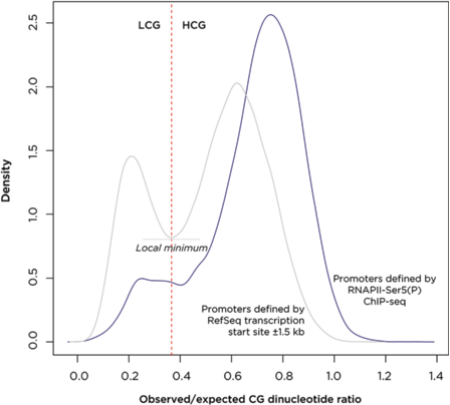
**RefSeq promoter annotations are over-represented for those with low-CG content compared with promoters used** ***in vivo*** **.** We reproduced a prior approach to categorize promoters based on observed/expected CG dinucleotide frequencies [21], showing the bimodal distribution (gray) that allows categorization into low-CG (LCG) and high-CG (HCG) groups. ChIP-seq to localize RNAPII-Ser5 (P) was used to define promoters used *in vivo* in unexposed, control HEK 293 T cells. The same bimodal CG dinucleotide distribution was observed (blue) but with a substantially lower proportion of LCG promoters, suggesting that the RefSeq annotation may include some inaccurate promoter predictions within this LCG subcategory. CG, cytosine-guanine dinucleotide; RNAPII-Ser5 (P), serine 5-phosphorylated RNA polymerase II.

The surprisingly limited transcriptional effect of massive pharmacologically mediated demethylation of the genome is consistent with previous studies [9]. Our focus turned to asking why only a subset of loci was especially prone to demethylation-induced transcriptional consequences. A function for DNA methylation is proposed to be to suppress transcriptional noise in the genome by silencing cryptic promoters [15]. To identify promoters in HEK 293 T cells and any cryptic promoters unmasked by the demethylation following 5-aza-CdR treatment, we used chromatin immunoprecipitation followed by massively parallel sequencing (ChIP-seq) for serine 5-phosphorylated RNA polymerase II (RNAPII-Ser5 (P)), which defines promoter regions [20] (Additional file 1: Tables S3 and S4). To assess the quality of these ChIP-seq promoter predictions, we measured their CG dinucleotide composition. Promoters in the human genome have been shown to be in two categories depending on their observed/expected CG dinucleotide frequencies [21]. Using the same approach as originally described but using the current RefSeq annotation, we could reproduce the bimodal distribution of high-CG (HCG) and low-CG (LCG) content (Figure 4) at the 3-kb flanking RefSeq transcription start sites. The same test of observed/expected CG dinucleotide frequencies for these loci occupied by RNAPII-Ser5 (P) in untreated HEK 293 T cells reveals a substantial shift towards the HCG category (Figure 4). As high-CG density is a feature long used to predict promoter locations [22], we interpret the enrichment of HCG content at sites of RNAPII-Ser5 (P) as supportive of the quality of the ChIP-seq predictions and indicative of the possibility that the LCG promoters defined by RefSeq gene annotation data are enriched for a subset of false-positive promoter predictions.

**Figure 5:**
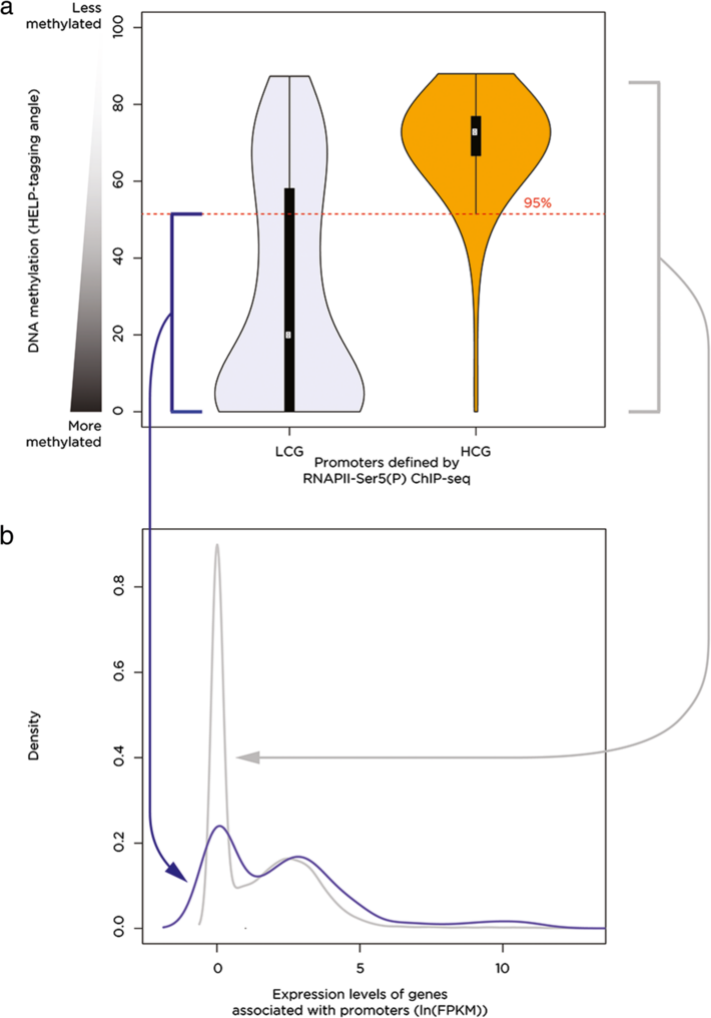
**RNA polymerase II is located at a subset of transcriptionally active, methylated LCG promoters.** When we study the LCG and HCG promoters defined by RNAPII-Ser5 (P) from Figure 4, we find that while HCG promoters are relatively uniformly less methylated (greater HELP-tagging signal, panel **(a)**, orange), the LCG promoters have what appears to be a bimodal distribution, with a substantial proportion showing higher DNA methylation (lower HELP-tagging signal, blue). By taking the group of promoters below the red dashed line (a) and comparing their expression (panel **(b)**, blue) with the genome-wide pattern (gray), we see that the genes associated with these promoters are comparably active to those throughout the genome as a whole (density plot of FPKM values). LCG, low-CG dinucleotide observed/expected ratio; HCG, high-CG dinucleotide observed/expected ratio; RNAPII-Ser5 (P), serine 5-phosphorylated RNA polymerase II; FPKM, fragments per kilobase per million reads.

When we studied the DNA methylation patterns at the RNAPII-Ser5 (P)-defined promoters in untreated HEK 293 T cells, those in the HCG category exhibited generally uniform DNA hypomethylation, whereas the smaller LCG subgroup included a substantial proportion of loci where the RNA polymerase is located at methylated DNA. We defined this subset of promoters as those with DNA methylation values less than the lower 95% confidence interval for HELP-tagging values of the HCG promoters (Figure 5a) and tested the expression levels of those genes relative to the genome as a whole. The fragments per kilobase per million reads (FPKM) values for this subgroup of genes with RNAPII-Ser5 (P) at methylated loci in untreated cells indicate that these genes are expressed at levels at least comparable to the remainder of the genes in the genome (Figure 5b). This is an unexpected association of a mark for active transcription start sites (RNAPII-Ser5 (P)) with a modification thought to be universally repressive at promoters (DNA methylation), with indications of active expression of the associated genes.

**Figure 6:**
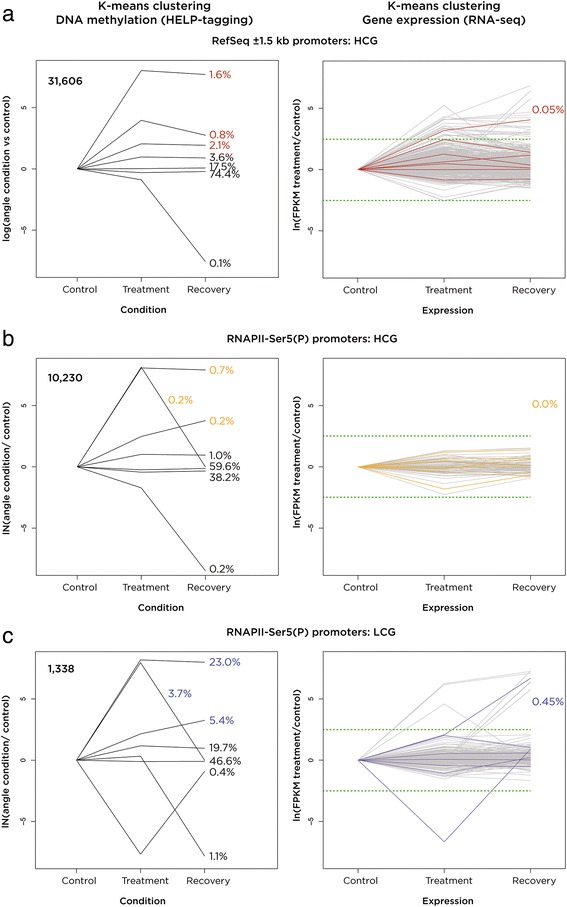
**Limited transcriptional consequences of promoter demethylation by 5-aza-CdR.** We show in panel **(a)** the RefSeq-defined HCG promoters and in panels **(b)** and **(c)** the RNAPII-Ser5 (P)-defined HCG and LCG promoters, respectively. On the left is a plot representing the k-means clustering of promoter methylation, with a very small proportion of promoters losing DNA methylation in both of the HCG categories (a, b) but a greater proportion of LCG promoters (c). When we take the genes associated with the k-means subgroups indicated by the colored percentages and represent their expression changes individually as gray lines and as subgroups depicted by colored lines, again reflecting the use of k-means clustering, we see very few genes to change expression levels, even though the proportion of promoters changing DNA methylation in panel (c) was relatively substantial. Percentages in the panels on the right represent proportions of all of the loci tested from the outset and are directly comparable with the percentages in the panels on the left. For example, while (23.0 + 3.7 + 5.4 =) 32.1% of the 1,338 promoters in panel (c) show loss of DNA methylation, when the expression of the associated genes is studied, only 0.45% of the 1,338 have induction of expression. LCG, low-CG dinucleotide observed/expected ratio; HCG, high-CG dinucleotide observed/expected ratio; RNAPII-Ser5 (P), serine 5-phosphorylated RNA polymerase II; FPKM, fragments per kilobase per million reads.

Having gained insights into the promoters used in the untreated cells, we then investigated the effects of 5-aza-CdR exposure at these loci. As we were concerned that the RefSeq-defined LCG promoters included a substantial proportion of false-positive predictions, we restricted our RefSeq-defined promoters to the HCG category as being more likely to be accurate. We tested the two groups of LCG and HCG promoters defined by RNAPII-Ser5 (P) ChIP-seq as a high-confidence reference standard. We measured the DNA methylation changes and associated RNA-seq expression differences at these three promoter categories. A k-means clustering approach allowed us to look for subsets of loci with transient or ‘imprinted’ changes in promoter methylation, looking for seven clusters, one in which no changes occur, and three each for gain and loss of DNA methylation, reasoning that this would allow us to discriminate between early, late, and transient changes between the acute and recovery stages. This analysis revealed a minority of promoters with these types of DNA methylation changes at the three categories of promoters (Figure 6), with most effects noted at the RNAPII-Ser5 (P) LCG category and minimal effects at both HCG promoter groups. When we selected those promoters with the greatest losses of DNA methylation in each category and studied the associated gene expression changes, we found negligible proportions of genes to be manifesting a transcriptional response (Figure 6). We conclude that even with careful promoter annotation, categorization into HCG and LCG subtypes, and detection of DNA methylation changes at a substantial proportion of ChIP-seq-defined RNAPII-Ser5 (P) promoters, transcriptional consequences of promoter demethylation are very rare events.

### Effects of DNA demethylation within bodies of genes

**Figure 7:**
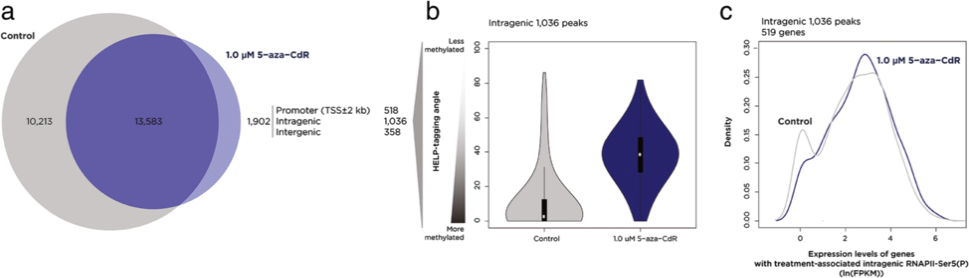
**Induction of RNAPII-Ser5 (P) by 5-aza-CdR in intragenic loci is associated with activation of silenced genes.** In panel **(a)**, we show that while the majority of ChIP-seq peaks following 5-aza-CdR exposure already were present in the untreated cells, the majority of the 1,902 new peaks found map to intragenic loci (defined as the RefSeq gene annotation excluding the ±2 kb around the transcription start site, used to define the promoter). The new 1,306 intragenic peaks are found to decrease DNA methylation (increase HELP-tagging signal, panel **(b)**) and are associated with 519 genes, whose expression before (gray) and after (blue) 5-aza-CdR treatment is shown using a density plot **(c)**. A decrease in the proportion of completely silenced genes (left peak) is observed in the genes gaining intragenic RNAPII-Ser5 (P), suggesting the activation of intragenic promoters. 5-aza-CdR, 5-aza-2′-deoxycytidine; RNAPII-Ser5 (P), serine 5-phosphorylated RNA polymerase II; FPKM, fragments per kilobase per million reads.h.

As global demethylation targeting euchromatin might be expected to have consequences in terms of unmasking of alternative intragenic promoters [23], we explored whether treated cells had evidence of such events, manifesting by the emergence of new RNAPII-Ser5 (P) ChIP-seq peaks. We found the peaks for RNAPII-Ser5 (P) to be almost completely concordant between control and 1.0 μM 5-aza-CdR-treated cells (Figure 7a). There was, however, a subset of new peaks in the treated and demethylated cells. At these new peak locations, we found a strong shift towards decreased DNA methylation (Figure 7b). RNA-seq is not an ideal test for novel promoter use but might be expected to show an overall expression increase in the situation of the activation of an additional cryptic promoter. When we tested the genes where new RNAPII-Ser5 (P) peaks were occurring in treated cells, we found evidence for a reduction in the proportion of completely silent genes (Figure 7c), indicating that these intragenic peaks may be involved in activating previously repressed genes. A total of 76 genes were found to have new intragenic RNAPII-Ser5 (P) peaks and induction of expression. This group of genes, when analyzed using Gene Set Enrichment Analysis (GSEA) [24], were observed to be significantly enriched for targets of H3K27me3 during differentiation [25] or Polycomb proteins [25,26] (Additional file 1: Table S5).

**Figure 8:**
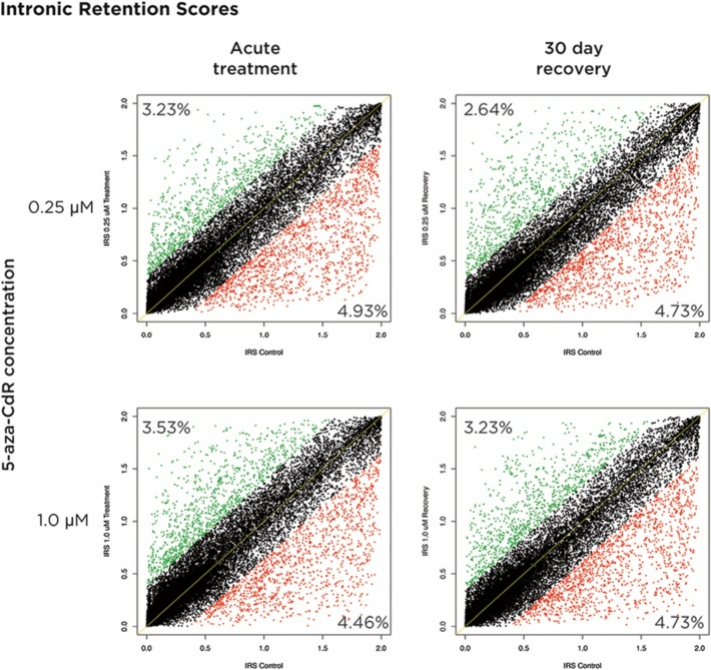
**No effect on primary transcript levels was found with 5-aza-CdR exposure.** The Intronic Retention Score (IRS) was calculated for each condition, with the loci changing 2× the standard deviation depicted by green (increased) or red (decreased) as a response to the drug exposure. No systematic shift to increased IRS with drug exposure was found, a finding that makes it unlikely that the primary RNA transcript is influenced by gene body demethylation by 5-aza-CdR. 5-aza-CdR, 5-aza-2′-deoxycytidine.

DNA methylation within the bodies of genes has also been implicated as a regulator of co-transcriptional splicing events, recruiting MeCP2 for exon recognition [23], suggesting that demethylation of transcribed gene bodies may have consequences for splicing and RNA processing. As mentioned earlier, we found the majority of genes changing expression with 1.0 μM 5-aza-CdR treatment to be loci annotated to have multiple isoforms, so altered splicing is a possibility at these genes, but these differentially expressed genes are so limited in proportion that this cannot be said to provide evidence for a systematic effect. We tested the alternative possibility that there may instead be measurable changes in the processing of the primary transcript. DNA methylation within a transcribed sequence has been associated with changing the rate of passage of RNA polymerase through the body of the gene [27], raising the possibility that the extensively demethylated gene bodies in our treated cells would be accompanied by increases in the amount of primary transcript. The RNA-seq assay we used is not restricted to the sequencing of processed mRNAs, allowing us to detect and quantify primary transcripts present. To quantify the amount of primary transcript, we calculated the Intronic Retention Score [28] as a measure of the degree to which the primary transcript was being sequenced in treated compared with control cells. Our expectation was that an increased speed of polymerase in demethylated genes would result in a skewing of Intronic Retention Scores (IRSs), with an increase in 5-aza-CdR-treated cells. We show in Figure 8 that there is no such skewing, arguing against the direct role of DNA methylation in RNA polymerase slowing and instead supporting the role of trimethylation of lysine 36 of histone H3 (H3K36me3) as an associated mark in transcribed gene bodies that is more likely to mediate polymerase slowing [28].

### Characteristics of subset of protein-coding genes with transcriptional changes

**Figure 9:**
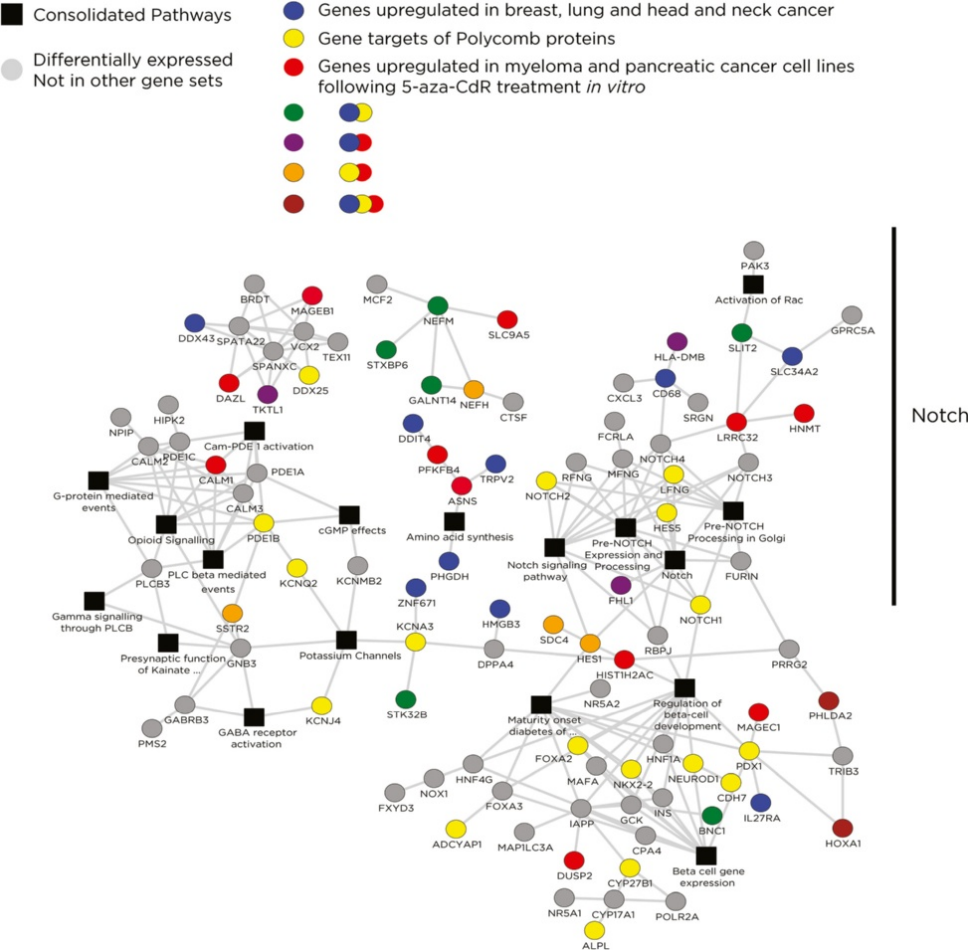
**The genes affected by 5-aza-CdR are comparable in multiple cell types and represent targets of Polycomb in ES cells.** A depiction of the interactions of the genes changing expression in response to 1.0 μM 5-aza-CdR (large circles of any color) with genes induced to express in myeloma cells (blue) and a highly overlapping set of genes induced to express by 5-aza-CdR in pancreatic cancer cells and genes up-regulated in breast cancer (orange), as well as genes that represent targets of Polycomb-mediated repression in ES cells (yellow). Genes involved in multiple gene sets combine those colors. These results indicate that our results in HEK 293 T cells may represent genomic responses in human cells more generally and suggests a mechanism based on reversing Polycomb effects that may mediate the drug’s therapeutic benefits in MDS. 5-aza-CdR, 5-aza-2′-deoxycytidine.

Finally, we turned our focus to the limited subset of genes with robust expression changes as a response to 5-aza-CdR (Figure 3). To understand the characteristics of the small subset of genes induced to change expression with 5-aza-CdR treatment, we performed GSEA on the genes differentially expressed in the 1.0 μM 5-aza-CdR-treated cells and found significant enrichment of several pathways (Additional file 1: Table S6). Three gene sets predominated. In common with the more limited group of genes with induction of expression associated with *de novo* intragenic promoters, one gene set reflected Polycomb-targeted loci in human embryonic stem (ES) cells [25] and genes marked with H3K27me3 in pluripotent cells [26]. A second group of genes is those that have previously been shown to be induced following *in vitro* exposure to 5-aza-CdR of human pancreatic [29] and multiple myeloma [30] cancer cell lines, indicating a common set of genes transcriptionally altered by 5-aza-CdR in our non-malignant cells that are also targeted by the drug in cultured malignant cells. The third group is a group of genes previously found to be overexpressed in several epithelial cancers [31-33], with the Notch pathway a common functional intersection for these gene groups. We illustrate the network connectivity and the different pathway representations of the genes involved in Figure 9.

## Discussion

With increasing interest in the use of epigenetically active drugs to ameliorate human diseases, it is valuable to define how these drugs work on a genomic level, an area of research that has been relatively under-explored to date, especially in non-malignant cells. We confirm previous observations that 5-aza-CdR is extremely powerful in demethylation of the genome [3], with the novel observation that the demethylation is targeting gene-rich, transcriptionally active euchromatin. We also observe a long-lasting imprint in cells no longer exposed to the drug, which has previously been found to occur at the majority of loci in the genome of HCT116 cells treated with 0.3 μM of 5-aza-CdR, with the demethylation imprint still present 42 days after drug exposure [34]. This is of interest as a potential model of acquired resistance to an epigenetically active drug - if a locus is already demethylated, it has become refractory to further demethylation. In spite of the disproportionate targeting of euchromatin, our data are consistent with prior reports indicating that transcriptional effects are limited to a small subset of protein-coding genes, with a similar effect on lncRNAs. This is despite the measurable demethylation of RefSeq and RNAPII-Ser5 (P)-defined promoters and the emergence of new promoters defined by loci acquiring RNAPII-Ser5 (P). A clue to the mechanism of transcriptional activation of 5-aza-CdR comes from the discovery that the intragenic acquisition of RNAPII-Ser5 (P) appears to be associated with the activation of previously silenced genes, possibly supporting a model of limited cryptic promoter activation [35]. The fact that we do not observe substantial gene activation is unexpected, as the common belief is that DNMT1 inhibitors should have this property. It may be significant that many previous studies have used malignant cells whereas we are using transformed HEK 293 T cells, which are not derived from a primary cancer.

In defining promoter categories, we made the unexpected discovery that the previous definition of LCG promoters [21] appears to over-represent those used *in vivo*, based on a comparison with those defined by ChIP-seq data for RNAPII-Ser5 (P). As the majority of the genome is depleted in CG dinucleotides, a random sampling of the genome included in this kind of analysis would relatively inflate the LCG proportion, suggesting that RefSeq-defined promoters in the LCG category should be viewed as annotations of questionable reliability. Another finding of interest about promoters was that RNAPII-Ser5 (P) can be located at methylated DNA and that the associated genes show patterns of expression that appear comparable to those in the remainder of the genome. This is a puzzling observation. One concern with any study that finds co-localization of genomic regulatory events is that co-localization does not necessarily imply the presence of the two events on the same alleles in the population of cells being tested. Scrutinizing the HELP-tagging data of Figure 5, it is clear that a substantial proportion of the readings are at loci of intermediate methylation, which supports a model of mixed allelic populations, allowing the RNAPII-Ser5 (P) to be present on a subset of alleles that are unmethylated. Further work will be necessary to resolve this unexpected finding.

The effect of pharmacological demethylation on promoters and transcription was limited and frequently uncoupled - promoters with methylation changes did not necessarily change the expression status of the associated gene. It has already been shown that some promoters can remain occupied by nucleosomes following pharmacological demethylation [13], and it is also possible that the lack of cognate transcription factors may be a reason why the acquisition of permissiveness, in terms of DNA methylation, is not accompanied by the induction of gene expression. The results of this study serve to emphasize that transcriptional regulation is a complex system that cannot readily be interpreted in terms of a single component like DNA methylation.

The relationship of DNA methylation with transcription in gene bodies has been recognized for some time to be different from that at promoters, referred to as ‘the methylation paradox’ [36], possibly serving a role to repress cryptic intragenic transcription [23], although this role has been questioned [37]. DNA methylation is also linked to co-transcriptional splicing regulation [35] and to modulation of the rate of RNA polymerase passage through the transcribed region [27]. Together, these prior observations indicate that we should expect changes in qualitative aspects of gene expression when DNA demethylation is occurring and disproportionately targeting transcribed regions of the genome. While there is some evidence for *de novo* establishment of RNAPII-Ser5 (P) promoters within gene bodies associated with 5-aza-CdR exposure, with limited transcriptional consequences, little evidence exists for altered primary transcript processing. While there is some evidence for activation of a small number of cryptic intragenic promoters, the effect is so small that it argues against a major reason for DNA methylation being to suppress transcriptional noise, as previously proposed [15].

A goal of the project was to understand why 5-aza-CdR targeted certain loci preferentially. As should be predicted, loci that are heavily methylated before treatment are more likely to become unmethylated as a response to the drug, which leads to our being able to define euchromatin as the major target compartment, as well as specific promoter subtypes that are more likely to be methylated. Unexpectedly, we found evidence that the genes that we found to change expression in HEK 293 T cells are significantly similar to those changing expression in malignant multiple myeloma and pancreatic cells exposed to 5-aza-CdR *in vitro* [29,30]. This indicates that there is a common set of human genes that respond to 5-aza-CdR, suggesting that the data we generated in HEK 293 T cells may be more broadly applicable to other human cell types. The genes induced to express show a strong enrichment for those implicated by Ben-Porath and colleagues [25] to represent the genes at which Polycomb-mediated silencing in ES cells is targeted. A model for cancer formation involving Polycomb proposes that silencing to create a pluripotent stem-cell-like epigenetic pattern is part of the induction of a self-renewal program that favors neoplasia [38]. The preferential demethylation and gene activation by 5-aza-CdR at targets of Polycomb-mediated silencing represents a tenable model for the drug’s therapeutic effects in MDS, consistent with the emerging body of evidence that MDS has a stem cell origin [39]. Set against this potential therapeutic benefit is the concern that 5-aza-CdR targets euchromatin, where associated chromosomal breakage would be directed towards the most gene- and transcriptionally enriched genomic compartment. This issue raises concerns about the risk:benefit ratio with DNMT1 inhibitor use in human disease, which may be justifiable in cancer treatment, as currently permitted, but may be more difficult to justify in less life-threatening disorders involving epigenetic changes.

## Conclusions

The use of powerful genome-wide assays to study the effects of pharmacological DNMT1 inhibition has confirmed several prior findings, including limited transcriptional effects despite profound global demethylation. New findings include the observation that demethylation can persist as an epigenetic imprint following drug withdrawal and cell recovery. In addition to the limited effects of demethylation by 5-aza-CdR on protein-coding genes, we see similar limited effects on lncRNAs and primary RNA transcript processing. This is an unexpected finding given the multiple prior reports of transcriptional activation following DNMT1 inhibitor treatment, but we note that the HEK 293 T cells that we study are not derived from malignant cells as are typically studied, potentially helping to explain this difference. Our use of ChIP-seq to define promoters reveals some potential limitations to current genomic annotations of transcriptional start sites and some new insights about the CG dinucleotide content and DNA methylation patterns at these *in vivo* promoters. We find evidence that the limited effects on transcription of protein-coding genes in HEK 293 T cells may involve activation of intragenic promoters, while similarities observed in the genes induced to express in our study with those in other human cell types treated in the same manner suggest that our results may apply to other cell types. We find support for 5-aza-CdR having effects that reverse Polycomb-mediated silencing, suggesting a mechanism for its therapeutic effect in MDS. While HEK 293 T cells are not derived from malignant cells, neither are they primary, untransformed cells, so we have to be cautious about interpreting how our findings translate to other cell types, but the preferential targeting of euchromatin for demethylation raises the concern that 5-aza-CdR may also have clastogenic properties, requiring caution in the use of this drug clinically for non-cancer conditions involving epigenetic dysregulation.

## Methods

### Cell culture and 5-aza-CdR treatment

We chose to work with the widely available and well-characterized HEK 293 T transformed, non-malignant cells, in order to avoid any bias that passage number could introduce in the analysis. HEK 293 T cells were grown in DMEM supplemented with 10% fetal bovine serum and 2 mM penicillin-streptomycin at 37°C in 5% CO_2_. 5-aza-CdR was dissolved in water to a final concentration of 10 mg/mL and stored in aliquots at −20°C. The 5-aza-CdR treatment was optimized to establish a working concentration, using a range from 0.25 to 2.0 μM. The cells were exposed to 5-aza-CdR for 3 days to allow the drug to be incorporated into DNA. Tissue culture medium was changed every day for both control and treated cells, to maintain the drug stability during treatment.

HEK 293 T cells were grown in triplicates for each condition, including a control group with no drug exposure. To allow recovery of the cells after the 3-day treatment, we maintained the treated cells in culture with fresh media lacking drug. Cells were passaged only when reaching 80% to 90% confluence, for a total period of 1 month. Cells were washed before trypsinizing to ensure that only adherent, viable cells were passaged. DNA, RNA, protein, and cross-linked chromatin were extracted from the same cell batch for each condition.

### Cell metabolism assay

Cells were seeded in 96-well plates using the same experimental conditions as for the 5-aza-CdR experiments. At the end of each time point, the WST-1 assay (Clontech) was performed following the manufacturer’s recommendations. Absorbance reads were registered after 40 min of incubation for 16 replicates at each time point and normalized by the number of cells present to quantify the net metabolic activity of the cells in culture. A total of 12 replicates were performed.

### Sample preparation

DNA, RNA, and cross-linked chromatin were extracted from the same cell batch for each condition. For DNA extraction, cells were re-suspended in lysis buffer (10 mM Tris EDTA (TE), 150 mM ethylenediaminetetraacetic acid (EDTA), and 1% sodium dodecyl sulfate (SDS)) supplemented with 10 mg/mL of RNase A and 20 mg/mL of Proteinase K and incubated at 50°C overnight. The lysed cells were phenol-chloroform extracted, and the resultant DNA was dialyzed in 0.2× SSC buffer (300 mM NaCl and 3 mM Na_3_C_6_H_5_O_7_, ph 7.0) for 24 h. The DNA sample was concentrated in the dialysis bags using polyethylene glycol, following which the quality and concentration of the DNA were measured by NanoDrop spectrophotometry. RNA was extracted using TRIzol (Invitrogen) using the manufacturer’s protocol. The quality and integrity of the RNA were measured using NanoDrop spectrophotometry and Bioanalyzer (Agilent).

For chromatin, cells were processed using the Myers Lab ChIP-seq protocol [40]. The untreated and 1.0 μM acutely treated cells were cross-linked in culture media with 1% formaldehyde for 10 min, quenching with 0.125 M Glycine. The cells were then washed with cold PBS, collected by centrifugation at 2,000 rpm for 5 min at 4°C and re-suspended in cold lysis buffer (5 mM PIPES (pH 8.0), 85 mM KCl, 0.5% NP-40, supplemented with fresh protease inhibitor cocktail (Roche)). Following collection, the crude nuclear preparation was re-suspended in 300 μL of cold radioimmunoprecipitation assay (RIPA) buffer (1× PBS, 1% NP-40, 0.5% sodium deoxycholate, 0.1 SDS, supplemented with fresh protease inhibitor cocktail (Roche)) and processed in a Bioruptor at the high setting. HEK 293 T cells were sonicated for a total of 15 min, in cycles of 30 s on/30 s off. The sonicated mixture was spun at 16,000 × *g* for 15 min at 4°C, and the chromatin was collected from the supernatant. The sample volume was brought to 1 mL with RIPA buffer, and 100 μL was saved as an input control.

### Luminometric methylation assay (LUMA)

To quantify global DNA methylation changes, LUMA analysis was performed [16]. For each time point, each of the DNA samples was digested in triplicate with 20 U EcoRI and either MspI or HpaII at 37°C overnight, purified and submitted to our institutional Genomics Core Facility for pyrosequencing. The percentage of methylation was calculated by the ratio of the incorporated (C + G) nucleotides after the HpaII digestion compared with the MspI digestion and normalized to the values obtained by EcoRI digestion ((A + C)/2).

### HELP-tagging assay

As previously described [41], 1 μg of DNA was used to generate HELP-tagging libraries. The indexed adapters used are listed in Additional file 1: Table S7. Genomic DNA was digested with either MspI or HpaII at 37°C overnight, purified and ligated to the first pre-annealed TruSeq-indexed Illumina adapters containing the T7 promoter sequence, as well as the EcoP15I recognition site (AE adapters [41]). After ligation, the DNA samples were digested with EcoP15I at 37°C overnight, end-filled, 3′ terminal A extended and ligated to the second pre-annealed Illumina adapter (AS adapter). Samples were then *in vitro* transcribed using the MEGAshort kit (Ambion), followed by retrotranscription (SuperScript III kit, Invitrogen) before amplification. Libraries were multiplexed for 50 bp single-end sequencing on the Illumina HiSeq 2500 platform at the institutional Epigenomics Shared Facility.

### Directional RNA-seq assay

DNase-treated, rRNA-depleted (Ribozero, Epicentre) RNA was used as a template for SuperScript III first-strand cDNA synthesis (Invitrogen), using oligo-dT as well as random hexamers. Actinomycin D was added to the reaction to prevent any possible amplification from contaminating genomic DNA. During second-strand synthesis, a dU/VTP mix was used to create directional libraries. Before library preparation, cDNA samples were Covaris-fragmented to 300-bp fragments. The samples were then end-filled, 3′ terminal A extended and ligated to pre-annealed TruSeq-indexed Illumina adapters. Uracil-DNA-glycosylase (UDG) treatment preceded the PCR reaction to amplify exclusively the originally oriented transcripts. Libraries were amplified using P5 and P7 Illumina primers and gel-extracted for size selection and primer-dimer removal. Before sequencing, libraries were tested using the BioAnalyzer to assure library quality, in terms of size and primer-dimer depletion. Indexed libraries were multiplexed for 100-bp single-end sequencing on the Illumina HiSeq 2500 platform at the institutional Epigenomics Shared Facility. The indexed adapters used are listed in Additional file 1: Table S8. Given the concordance of extremely limited effects on transcription for different time points and drug dosages, and following recommendations of ENCODE for RNA-seq experiments [19], we allowed the four treatment conditions to act as biological replicates, increasing confidence in our findings of minimal transcriptional effects of 5-aza-CdR.

### ChIP-seq assay

Chromatin immunoprecipitation was performed using 3 × 10^6^ cells using the Myers Lab ChIP-seq protocol [40]. HEK 293 T cells were crosslinked in culture media with 1% formaldehyde for 10 min, quenching with 0.125 M Glycine. The cells were then washed with cold PBS, collected by centrifugation at 2,000 rpm for 5 min at 4°C, and resuspended in cold Farnham lysis buffer (5 mM PIPES (pH 8.0), 85 mM KCl, 0.5% NP-40, supplemented with fresh protease inhibitor cocktail (Roche)). Following collection, the crude nuclear preparation was resuspended in 300 μL of cold RIPA buffer (1× PBS, 1% NP-40, 0.5% sodium deoxycholate, 0.1 SDS, supplemented with fresh protease inhibitor cocktail (Roche)) and processed in a Bioruptor at the high setting. HEK 293 T cells were sonicated for a total of 15 min, in cycles of 30 s on/30 s off. The sonicated mixture was spun at 16,000 × *g* for 15 min at 4°C, and the chromatin was collected from the supernatant. The sample volume was brought to 1 mL with RIPA buffer, and 100 μL was saved as an input control. Magnetic beads (Invitrogen) were washed three times with 5 mg/mL BSA in PBS and supplemented with freshly added protease inhibitors. RNAPII-Ser5 (P) antibody (5 μg, Active Motif catalog number #61085) was added to the bead slurry, incubating the mixture overnight at 4°C. The antibody-coupled beads were washed three times with PBS/BSA, added to the chromatin sample, and incubated in a rotor at 4°C overnight.

After immunoprecipitation, beads were collected by magnetic separation and washed five times with cold LiCl Wash buffer (100 mM Tris–HCl pH 7.5, 500 mM LiCl, 1% NP-40, 1% sodium deoxycholate). After a final wash with cold 1× TE buffer, the beads were resuspended in 200 μL IP elution buffer (1% SDS, 0.1 M NaHCO_3_, containing proteinase K and 0.2 M NaCl), and both the immunoprecipitated and input samples were de-crosslinked at 65°C overnight. ChIP products were purified with the DNA Clean and Concentrator kit (Zymo) and eluted in 60 μL of elution buffer. The efficiency of the ChIP was tested by enrichment quantification of immunoprecipitated/input DNA ratios at candidate positive loci compared with those at negative regions, using real-time quantitative PCR (RT-qPCR). The primers used are presented in Additional file 1: Table S9. For library preparation, the samples were end-filled, 3′ terminal A extended and ligated to pre-annealed TruSeq-indexed Illumina adapters. The indexed adapters used are listed in Additional file 1: Table S10. Libraries were amplified using P5 (5′-AATGATACGGCGACCACCGA-3′) and P7 (5′-CAAGCAGAAGACGGCATACGAGAT-3′) Illumina primers and gel extracted for size selection and primer-dimer removal. Before sequencing, the quality of the libraries was checked using the Agilent BioAnalyzer to confirm the correct size (250 to 500 bp) and primer-dimer depletion. Libraries were multiplexed and single-end sequenced on the Illumina HiSeq 2500 with 100-bp read length at the institutional Epigenomic Shared Facility.

### HELP-tagging data analysis

Sequencing reads were aligned by the WASP pipeline [42] version 3.1.4 (rev. 6598), using the CASAVA aligner from Illumina (ELAND 1.7.0). Transformation of raw sequence reads to the angle calculation previously described [41] was performed using an MspI reference from HEK 293 T cells and scaled to create a 0 to 100 range. Data analysis was performed using a bespoke pipeline in the R environment (version 2.15.0). A median of 4.5 million reads was obtained for each sample, with 98% of them passing filter and 76% aligning to the reference genome.

### RNA-seq data analysis

For RNA-seq analysis, sequencing reads were aligned by the WASP pipeline [42] version 3.1.3 (rev. 6589) using *gsnap* (2012-07-20) [43]. *Cufflinks* tools [18] version 2.1.1 were then used to assemble aligned RNA-seq reads into transcripts, estimate their abundances, and test for differential expression and regulation [18]. For each sample, 15.5 million reads were obtained on average, 89% of them passing filter and 93% aligning to the reference genome. *Cufflinks* was used to calculate the score of FPKM for each transcript. To generate reference genomes, GTF files were obtained from the UCSC Genome Browser. We used the *Cuffdiff* package [18] to compare FPKM values and obtain a list of differentially expressed genes. k-Means clustering was used to group RefSeq gene expression levels. For the estimation of intronic retention, the IRS was calculated following the previously reported approach [28]. The formula employed is presented below:

$$ \mathrm{I}\mathrm{R}\mathrm{S}=2\times \frac{\varSigma \frac{\mathrm{intronic}\;\mathrm{coverage}}{\mathrm{intronic}\;\mathrm{length}}}{\varSigma \frac{\mathrm{exonic}\;\mathrm{coverage}}{\mathrm{exonic}\;\mathrm{length}}+\varSigma \frac{\mathrm{intronic}\;\mathrm{coverage}}{\mathrm{intronic}\;\mathrm{length}}} $$

By calculating the ratio between control and tested samples, and then considering the genes with a ratio greater than 2× the standard deviation, we obtained a list of the candidate genes with the most extreme intronic retention.

### ChIP-seq data analysis

ChIP-seq reads were aligned by the WASP Pipeline [42] version 3.1.5, using MACS [44] version 1.4.2 as the peak finder and *bowtie* [45] version 0.12.7 as the aligner. A median of 19 million reads was obtained for each sample, with 89% of them passing filter and 77% aligning to the reference genome. Data analysis was performed using a bespoke pipeline within the R environment (version 2.15.0).

### Self-organizing map analysis

To examine the relationships between five different genomic variables, we used an artificial neural-learning-based approach, the self-organizing map (SOM) [46]. We used 100-kb sliding windows with a step size of 50 kb as we have previously described [14]. To build the SOM, we used information from HELP-tagging data for control samples and the acute values for the 1.0 μM 5-aza-CdR-treated cells, the mean of gene expression levels of all RefSeq genes within the window, and the cumulative number of HpaII sites per window. All vectors were tagged by 5-aza-CdR response and whether they belonged to one of the five quartiles of DNA methylation and gene expression levels. After training, all data were re-introduced to the grid a final time and selected annotations revealed as a dual color intensity graph in order to examine the distribution of features. Overall clustering patterns in the data were also examined using a U-matrix representation of the grid, which represents a similarity graph where a linear grayscale is used to indicate how similar a node vector is to its immediate neighbors in vector space.

### Promoter categorization

Replicating a previously published approach [21], we categorized promoters based on CG dinucleotide content. A cutoff of 0.366 was established at the local minimum of the bimodal distribution for the observed/expected CG ratio of all RefSeq promoters (±1.5 kb flanking the TSS), while the cutoff for the RNAPII-Ser5 (P) promoters was 0.401.

### Public availability of data

All genome-wide data are available from the GEO resource at http://www.ncbi.nlm.nih.gov/geo/ under the accession number GSE62590.

## Additional file

Additional file 1:
**Supplemental tables.** Supplemental tables are provided with further experimental results and details about the primer and adapter sequences used.

## Abbreviations

5-aza-CdR: 5-aza-2′-deoxycytidine
5-aza-CR: 5-azacytidine
5mC: 5-methylcytosine
CG: CpG, cytosine-guanine dinucleotide
ChIP-seq: massively parallel sequencing of chromatin immunoprecipitated DNA
DNMT: DNA methyltransferase
EGCG: epigallocatechin-3-gallate
ES: embryonic stem (cells)
FPKM: fragments per kilobase per million reads
GSEA: Gene Set Enrichment Analysis
H3K36me3: trimethylation of lysine 36 of histone H3
HCG: high-CG dinucleotide observed/expected ratio
HSPC: hematopoietic stem and progenitor cell
ICF: immunodeficiency, centromeric region instability, facial anomalies (syndrome)
IRS: Intronic Retention Score
LCG: low-CG dinucleotide observed/expected ratio
lncRNA: long non-coding RNA
LUMA: Luminometric Methylation Assay
MDS: myelodysplastic syndrome
RNAPII-Ser5 (P): serine 5-phosphorylated RNA polymerase II
RNA-seq: massively parallel sequencing of ribonucleic acid (RNA)
SOM: self-organizing map
